# Supplementary material for: Causal interplay between lactose intolerance and gut microbiota: a combined bidirectional Mendelian randomization and in vivo validation study
Source: Front Nutr. 2026 Jun 1;13:1803337. doi: 10.3389/fnut.2026.1803337 (PMC13265576; doi:10.3389/fnut.2026.1803337)
Supplement: Supplementary file 1 [file Data_Sheet_1.zip › supplementary materials/Forward/forest plot/ebi-a-GCST90027744.finngen_R12_E4_LACTONAS.pdf]

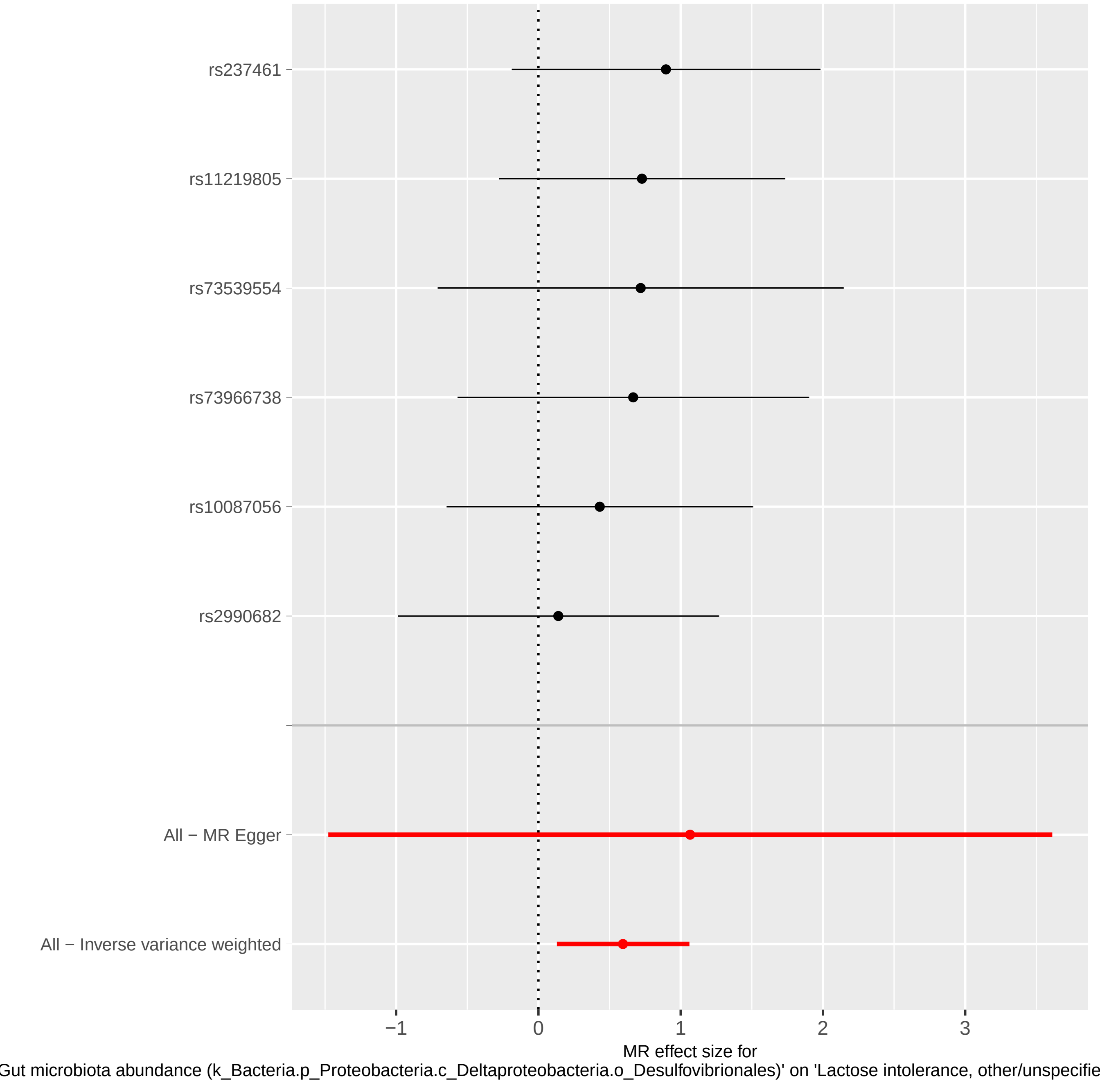
rs237461
rs11219805
rs73539554
rs73966738
rs10087056
rs2990682
All − MR Egger
All − Inverse variance weighted
−1
0
1
2
3
MR effect size for
'Gut microbiota abundance (k_Bacteria.p_Proteobacteria.c_Deltaproteobacteria.o_Desulfovibrionales)' on 'Lactose intolerance, other/unspecifie
